# Role of Surfactants in the Properties of Poly(Ethylene Terephthalate)/Purified Clay Nanocomposites

**DOI:** 10.3390/ma11081397

**Published:** 2018-08-10

**Authors:** Elaine Pereira dos Santos, Marcus Vinícius Lia Fook, Oscar Manoel Loureiro Malta, Suédina Maria de Lima Silva, Itamara Farias Leite

**Affiliations:** 1 Programa de Pós-Graduação em Ciência e Engenharia de Materiais, Universidade Federal da Paraíba, João Pessoa PB 58051-900, Brazil; elainesantosufpb@gmail.com; 2 Laboratório de Avaliação e Desenvolvimento de Biomateriais do Nordeste—CERTBIO, Unidade Acadêmica de Engenharia de Materiais, Universidade Federal de Campina Grande, Campina Grande PB 58429-900, Brazil; marcus.liafook@certbio.ufcg.edu.br; 3 Departamento de Química Fundamental, Universidade Federal de Pernambuco, Recife PE 50670-901, Brazil; olmalta@gmail.com; 4 Unidade Acadêmica de Engenharia de Materiais, Universidade Federal de Campina Grande, Campina Grande PB 58429-900, Brazil; suedina.silva@ufcg.edu.br; 5 Departmento de Engenharia de Materiais, Universidade Federal da Paraíba, João Pessoa PB 58051-900, Brazil

**Keywords:** PET, purified clay, surfactants, nanocomposites, properties

## Abstract

Purified clay was modified with different amounts of alkyl ammonium and phosphonium salts and used as filler in the preparation of PET nanocomposites via melt intercalation. The effect of this type of filler on morphology and thermal and mechanical properties of PET nanocomposites was investigated by X-ray diffraction (XRD), differential scanning calorimetry (DSC), thermogravimetric analyses (TG), tensile properties, and transmission electron microscopy (TEM). The results showed that the mixture of alkyl ammonium and phosphonium salts favored the production of PET nanocomposites with intercalated and partially exfoliated morphologies with slight improvement in thermal stability. In addition, the incorporation of these organoclays tended to inhibit PET crystallization behavior, which is profitable in the production of transparent bottles.

## 1. Introduction

The synthesis of new materials with optimized properties is an area in constant expansion in Materials Science. A significant advance in this area has occurred with the synthesis of polymer/layered silicates nanocomposites employing polymer from synthetic and renewable resources [1,2,3,4], in which the structural order of the material can be controlled at the nanoscale, resulting in improvements in various properties, among them tensile strength. This class of materials has attracted considerable interest from researchers and industry [5]. These materials are commonly prepared by three methods: melting, in situ, and solution evaporation. Among them, the melt intercalation method is the most attractive due to its low cost, high productivity, and compatibility with existing processing techniques [2,6,7]. However, the main limitation in melt processing is thermal decomposition of organic modifiers, particularly alkyl ammonium salts, when high temperatures are involved in the processing [8,9].

It is known that when PET nanocomposites are prepared by extrusion method, high temperature ranges of 280 °C are necessary, and can result in thermal decomposition of the alkyl ammonium ions commonly applied in organic modification of clays, which may change not only the interface between the charge and the polymer matrix, but also induce degradation of the polyester [8,10]. However, the chemical structure of the organic salt, such as length and number of alkyl chains, is also a determining factor in the thermal stability of the polymer nanocomposites [8,11,12,13,14,15].

Fornes and coworkers [16] examined the polymer degradation and color formation in polyamide 6 (PA6)/organoclay nanocomposites during melt processing. They showed that the kind of PA and the chemical structure of the surfactant were the two major factors affecting molecular mass decrease and color generation. Similar degradation phenomena were presented in polycarbonate/clay nanocomposites [17]. The degradation of PET/clay nanocomposites during melt extrusion was studied by Xu, et al. [18], who observed a significant reduction in PET molecular weight as a result of surfactant degradation of organoclay during processing. The level of degradation was found to be dependent on both the clay structure and surfactant chemistry. Alkyl phosphonium-based surfactants were thermally more stable than alkyl ammonium-based surfactants [19]. Likewise, Patro, et al. [20] compared these two surfactant types in PET/organoclay systems. They observed that the onset temperature of decomposition for phosphonium clays was higher than 300 °C, whereas for ammonium clays, it was around 240 °C, resulting in a significant decrease in the molecular weight and mechanical properties of PET during processing due to thermal degradation of ammonium surfactants. On the other hand, the molecular weight of PET was not considerably reduced during processing upon addition of phosphonium clay.

Therefore, organophilic clays prepared with thermally stable organic modifiers for use in nanocomposites are necessary and have been reported in the literature [13,14,15,19,21,22]. According to Shah and Paul [23], the degradation of organophilic clay can limit the extent of intercalation and/or exfoliation in nanocomposites. In order to overcome this limitation, Leite, et al. [13] showed that although the thermal stability of modified clays with alkyl ammonium salt can be improved by applying purification procedures, these are not yet sufficient for processing at elevated temperatures as, for example, 260 °C.

In this study the mixture of alkyl ammonium and phosphonium salts was used in the organic modification of purified clay at different amounts and then incorporated into the PET polymer by melting method. It is believed that these organoclays can be good candidates for the preparation of nanocomposites using polymeric matrices that require high processing temperatures. This study then investigated the morphology, thermal and mechanical properties of PET hybrids.

## 2. Results and Discussion

**Figure 1:**
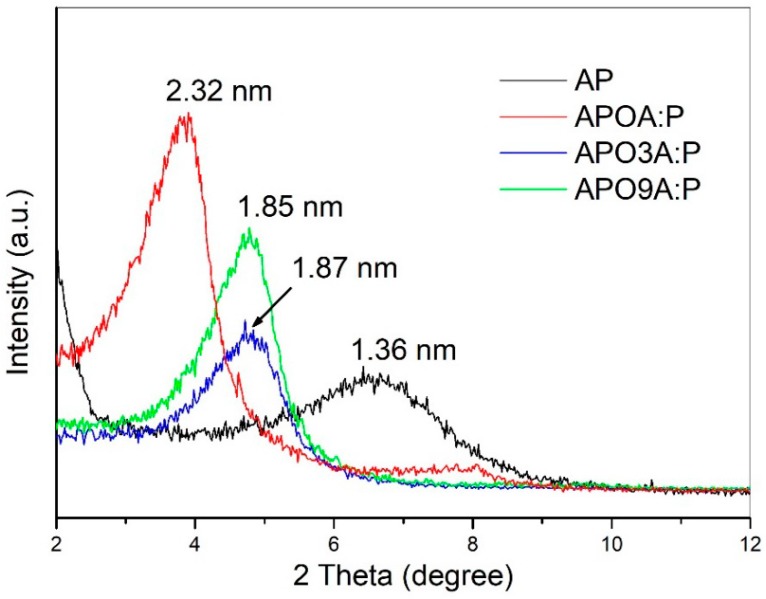
X-ray diffraction (XRD) patterns of the purified clay (AP) and organically modified purified clay with a mixture of surfactants A-P containing different amounts of alkyl ammonium (A) and alkyl phosponium (P).

**Figure 2:**
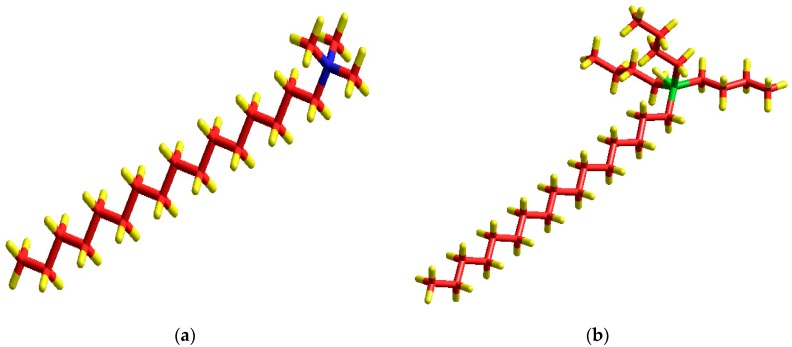
Molecular geometry of the surfactants: (**a**) alkyl ammonium (A) and (**b**) alkyl phosphonium (P). Blue is nitrogen, red is carbon, yellow is hydrogen, and green is phosphorous cylinders.

The X-ray diffraction (XRD) patterns of the purified clay as well as organically modified purified clay with a mixture of A-P surfactants containing different amounts of A and P are shown in Figure 1. The molecular geometry of the alkyl ammonium (A) and alkyl phosponium (P) surfactants are presented in Figure 2. The purified clay (AP) exhibited an interlayer spacing (d001) of 1.36 nm. After the organic modification of the purified clay with the A-P surfactant, an increase in the d001 from 1.36 nm (AP) to 2.32 nm (APOA:P), 1.87 nm (APO3A:P) and 1.85 nm (APO9A:P) was observed. The higher increase in d-spacing (70%) for APOA:P can be related to the steric hindrance and volume of the alkyl phosphonium surfactant that promoted a conformational disorder in the clay galleries [8,20]. This same behavior was observed by Torok, et al. [24], Calderon, et al. [25] and Leite, et al. [13], who observed a pseudo-trilayer arrangement when the alkyl phosphonium surfactant was employed in the pure form. It is believed that the higher value of basal spacing could facilitate the insertion of the polymer in the galleries of the organoclay and thus promote the intercalation, or even the exfoliation, of the polymer nanocomposite. The APO3A:P and APO9A:P samples, which were verified a d001 of approximately 1.86 nm, seem to display a bilayer structure [24,25]. This can be attributed to the higher levels of the alkyl ammonium in relation to the alkyl phosphonium surfactant, resulting in a lower steric effect and tending toward a more orderly state due to the lower volume alkyl ammonium surfactant (Figure 2).

It was also observed (Figure 1) that when the content of the alkyl ammonium surfactant (A) is increased as a function of amount of the alkyl phosphonium surfactant (P) in the A-P mixture, there is a gradual increase in the intensity of the diffraction peak (001) for APO3A:P and APO9A:P samples, respectively. In these samples higher levels of alkyl ammonium surfactant (A) predominate, which, because of their lower steric effect, tends toward a more ordered conformation as mentioned previously [8,13,15,16,18,26,27].

Regarding broadest diffraction peak (001), it was observed (Figure 1) that APO3A:P presents the highest broadest and lowest intensity of the samples. This may be indicative of a greater degree of disorder (gauche conformations) [13], possibly due to the volume of the cationic head (tributyl phosphonium). Evidence of this behavior can still be observed in the theoretical study of molecular modeling [12]. This observation is in accordance with the XRD data reported by Ammala, et al. [2].

In summary, it was observed that the intercalation of the surfactants in the AP clay using the mixture of both ammonium and phosphonium surfactants was successful, regardless of the amount of A and P surfactants involved in the mixture.

**Figure 3:**
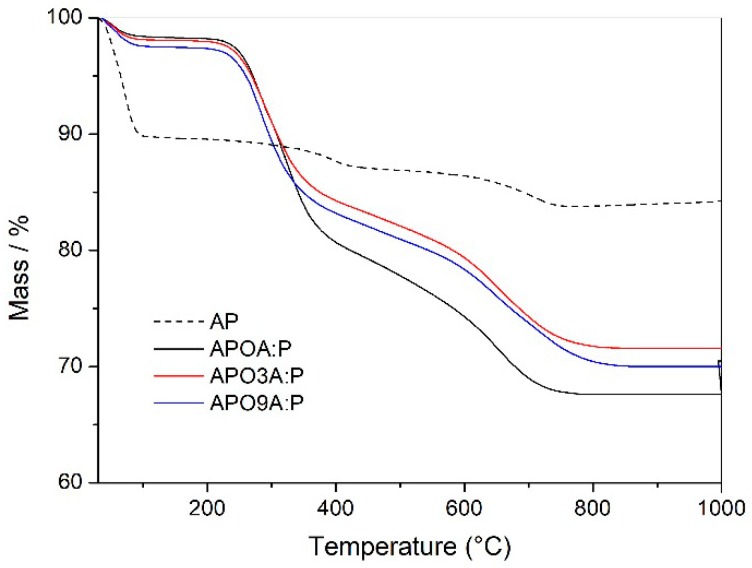
Thermogravimetric analyses (TG) curves of the purified clay (AP) and organically modified purified clay with a mixture of surfactants A-P containing different amounts of A and P.

Figure 3 shows thermogravimetric analyses (TG) curves for organobentonites and Table 1 presents the decomposition steps for these samples. The purified clay (AP) exhibited three thermal degradation transitions as shown in Table 1. The first one (T_H_2_O_) occurred at 71 °C and is attributed to the volatilization of both free water (i.e., the water sorbed on the external surfaces of crystals) and water inside the interlayer space, which forms hydration spheres around the exchangeable cations. The second transition (T_OH_) took place at 730 °C and is attributed to the loss of structural water resulting from clay dehydroxylation [2,28]. This clay also presented a third peak at 420 °C, which can be associated with residues from the purification process as evinced by Leite, et al. [13].

The amount of free water and interlayer water was appreciably reduced after organic modification of the purified clay with the surfactant A-P [29]. Considerable reduction was observed, especially for the APOA:P sample, consistent with the greater degree of hydrophobicity of the alkyl phosphonium ions present in higher amounts in this sample. However, the water volatilization temperature was also reduced.

Between 200 and 400 °C, the AP clay showed no change induced thermally. Therefore, the peaks in this region for all organophilic bentonite were attributed to the decomposition of the surfactant [30] in the range of 282–292 °C. The weight loss occurred in only one step, except for the APOA:P sample, which showed two steps of decomposition, one belonging to the A surfactant (274 °C) and the other to the P surfactant (302 °C). APO3A:P and APO9A:P organobentonites presented weight losses similar to (~16%) and smaller than the APOA:P (~19%) sample, indicating an improvement in the thermo-oxidative stability.

Based on these results, it is observed that thermo-oxidative stability is practically constant when the amount of surfactant A in the mixture A-P is increased in the purified clay (AP) in relation to the composition APOA:P, containing a ratio of 1:1 m/m, which was not expected. This behavior can be attributed to the high amount of surfactant A in the mixture A-P, which tends toward an ordered state with higher packing density, principally when the amount of surfactant P is reduced in the mixture of surfactant A-P.

Table 1 also shows practically the same values (~69%) of intercalation of the surfactant A-P in the galleries of clay (AP), except for the APO3A:P sample whose surfactant percentage was 61%. The carbonaceous residues formed at 260 °C increased slightly due to the increase in the surfactant A content in the mixture A-P for all organophilic bentonites. This slight increase shows a susceptibility to decomposition of the alkyl ammonium salt. Similar behavior has been observed by Guan, et al. [31], Leite, et al. [13] and Hedley, et al. [32]. Even so, it is verified that the thermo-oxidative stability of the different compositions of organophilic bentonites is not altered, as shown in Table 1.

According to these results, the introduction of varied contents of both surfactants A and P in the clay AP resulted in organobentonites with higher thermo-oxidative stability, especially the APO3A:P sample, whose Tmax value was 292 °C. Therefore, it can be inferred that these organophilic clays may be potentially useful in obtaining PET nanocomposites with considerable thermal properties.

**Figure 4:**
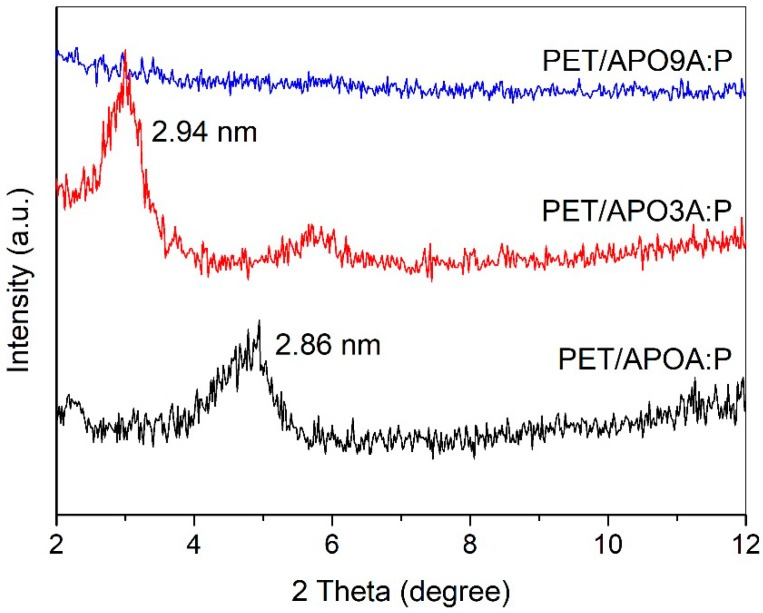
Diffractograms of PET hybrids with 1% by weight of modified AP clay with the mixture of surfactants A-P containing varying amounts of A and P: PET/APOA:P, PET/APO3A:P, and PET/APO9A:P.

The XRD patterns of the PET hybrids with 1% mass of clay AP modified with a mixture of surfactants A-P are shown in Figure 4. The incorporation of the organobentonites APOA:P and APO3A:P to the PET resulted in the obtaining of nanocomposites with predominantly intercalated morphology, sorted according to the presence of the basal reflections (001) of high intensity in the XRD pattern of hybrids. The basal spacing (d001) increased from 2.32 nm to 2.86 nm when the APOA:P clay was incorporated into the PET, and from 1.87 nm to 2.94 nm when the incorporation of APO3A:P clay happened to the referred polymer.

When the organoclay APO9A: P was incorporated into the PET, the characteristic peak of d001 of clay was not registered in the XRD pattern (Figure 4) suggesting the obtainment of nanocomposites with predominantly exfoliated morphology. Based on these results, it is evident that the clay AP organophilizated with the mixture of the surfactant A-P containing high levels of the surfactant A contributed to the greater intercalation of the PET chains between the clay layers.

**Figure 5:**
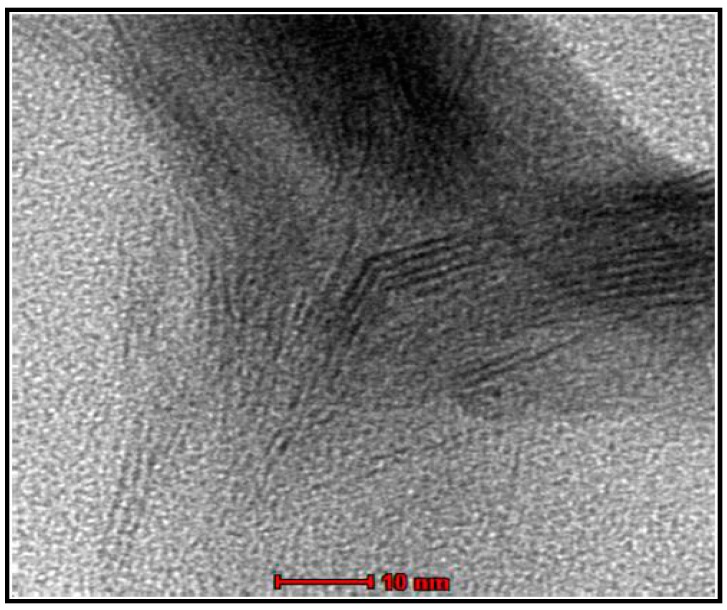
Transmission electron microscopy (TEM) image of PET hybrid containing 1% weight of clay APO9A:P.

In order to investigate the morphological variations suggested by the observations of XRD discussed above, transmission electron microscopy (TEM) was used and the micrograph of the hybrid PET containing 1% of weight of clay APO9A:P is shown in Figure 5. The light areas represent the PET matrix and dark areas represent the clay.

The micrograph of the hybrid PET/APO9A P (Figure 5) shows a partially exfoliated morphology composed of clay lamellae evenly distributed in the polymer matrix, with areas containing exfoliated lamellae and some areas with an interleaved structure. This result confirms the disappearance of the basal reflections (001) on the diffractogram of Figure 4, showing partial exfoliation in this composition.

**Figure 6:**
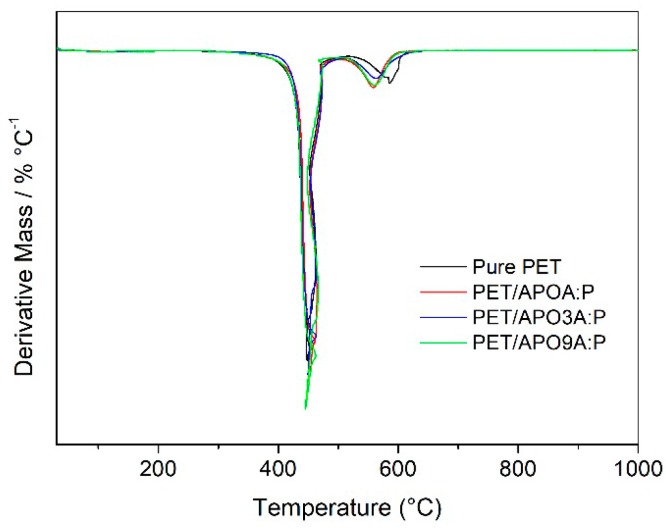
Derivative mass curves of pure PET and PET nanocomposites.

The thermo-oxidative stability of pure PET and PET nanocomposites containing 1% mass of modified AP clay with the mixing surfactants A-P was evaluated under air atmosphere. DTG curves of pure PET and PET nanocomposites are shown in Figure 6, and the data for weight loss are exhibited in Table 2.

The maximum decomposition temperature (T_max_) for PET nanocomposites did not reveal improvements in polyester thermo-oxidative stability when organophilic clays were introduced in the PET matrix. The percentage of carbonaceous residues in 600 °C increased in the range of 2 to 3% for all hybrids when compared to pure PET. These residues form a thermally insulating layer which protects the polymeric matrix, and still, due to the barrier effect, impedes the release of volatiles products generated during the decomposition [13].

It is observed that the decomposition temperature at 10% of weight loss recorded increases between 1 and 7 °C for all PET hybrids, especially for the samples PET/APOA:P and PET/APO3A:P. This is due to the high P concentrations in the mixtures 1:1 and 3:1 m/m of the A-P salt intercalated in the clays, resulting in higher decomposition temperature values and favoring a higher thermo-oxidative stability.

Improvements in the thermo-oxidative stability of polymeric nanocomposites are generally attributed to the creation of a tortuous path, resulting from the dispersion of clay and the slow diffusion of oxidative substances by the material [33]. According to the literature [34,35], the increase in thermal stability depends not only on the amount of clay but also on the quality of the clay dispersion and the type of structure formed in the nanocomposite.

In general, it was observed that the morphologies exhibited by intercalated nanocomposites PET/APOA:P and PET/APO3A:P promoted a slight increase in the values of thermo-oxidative stabilities. Moreover, it was verified that partially exfoliated nanocomposite morphology PET/APO9A:P did not lead to improvement in the thermo-oxidative stability compared to pure PET. This may have been promoted by degradation of the alkyl ammonium salt (A), which generally proceeds by Hoffman removing, present in greater quantity (A) in these samples. Similar behavior involving the degradation of alkyl ammonium salt is also reported in the literature [36,37].

The differential scanning calorimetry (DSC) results of pure PET and PET hybrids with 1% PA modified clay with a mixture of A-P salts under heating and cooling are shown in Table 3. It is noticed that the incorporation of different purified clay compositions (AP) to the PET polymer practically did not alter the thermal transitions, such as the glass transition temperature (Tg), crystallization temperature under heating (Tch), and crystalline fusion temperature (Tm) of samples. Likewise, under the cooling crystallization temperature (Tcc) of PET, the hybrid remained practically unchanged with the intercalation of organoclay (APO3A: P), except for the hybrid PET/APO9A:P, which crystallized at a temperature of 194 °C, slightly higher than the pure PET (192 °C). This indicates that the clay can act as a heterogeneous nucleation agent as observed by Wang, et al. [28]. Similar behavior has also been observed by Calcagno, et al. [29]. Concerning the degree of crystallinity (Xc), an increase from 7 to 12% was verified for all samples, especially for hybrid PET/APO9A:P when correlated to pure PET. The different values of the degrees of crystallinity (Table 3) may be associated with heterogeneity of the clay dispersion in the PET, resulting in different nucleating effects. This result is in agreement with the crystallization studies reported by Calcagno and collaborators [29].

**Figure 7:**
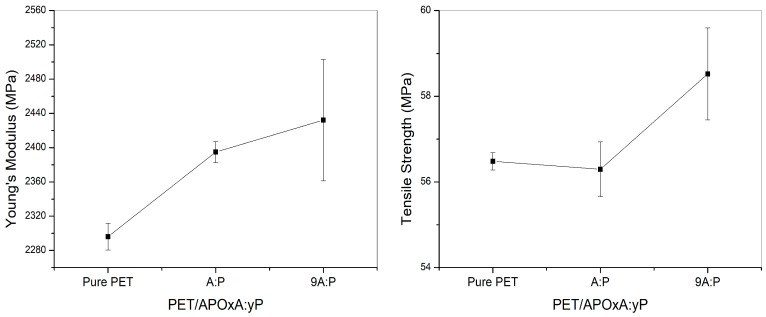
Young’s modulus curves and tensile strength of pure PET and PET hybrids containing 1% by weight of clays APOA:P and APO9A:P.

Figure 7 shows the curves obtained by tensile tests for pure PET and to PET hybrids containing 1% of mass clay modified AP with the mixture of the A-P salts. The incorporation of different compositions of organophilic clays in the PET matrix gradually increased the rigidity of the nanocomposites. Regarding tensile strength, it is noted that there was no significant changes to this property, regardless of the AP clay composition incorporated into the PET. It is believed that these properties can be improved since the nanocomposites are stabilized (additives) during processing. Chang and Park [30] observed that the increase in tensile property depends on the interactions between the polyester molecules and clay as well as its rigid nature. Furthermore, the clay is more rigid than the polyester molecules and does not deform or relax as the polymer molecules do.

## 3. Materials and Methods

### 3.1. Materials

The poly(ethylene terephthalate) (PET), BG1180-W, degree bottle, was supplied by Braskem (Bahia, Brazil) in the form of white color pellets, with an intrinsic viscosity 0.80 ± 0.02 dL/g (ASTM D4603-03), acetaldehyde content 1.0 ppm, melting point 245 ± 5 °C, and density of 1.39 g/cm^3^. It was used as polymer for preparing the nanocomposite. The Argel bentonite was purified with the purpose of removing organic matter, according to the procedure reported by Leite, et al. [13], and coded as AP. This montmorillonite (AP) was provided in powdered form, with particle size ≤ 45 μm by the Mining and Geology Laboratory of the Federal University of Campina Grande, and whose cation exchange capacity (CEC) was 92 meq/100 g, as determined by Phelps and Harris [38] method. It was used as filler in the PET nanocomposites preparation.

In this study, our goal was also to evaluate the influence of the surfactants in the morphology and thermal properties of the PET nanocomposites. The purified clay was then modified using the mixture of the both surfactants, cetyl trimethyl ammonium bromide (C_16_H_33_(CH_3_)_3_N^+^Br^−^) (A) (MM = 364.45 g/mol) and hexadecyl tributyl phosphonium bromide (C_16_H_33_(C_4_H_9_)_3_P^+^Br^−^) (P) (MM = 507.65 g/mol) in different amounts, supplied by Vetec (São Paulo, Brazil) and Sigma-Aldrich (São Paulo, Brazil), respectively.

### 3.2. Purified Montmorillonite Organic Modification

The purified montmorillonite (AP) was organically modified with the mixture of alkyl ammonium (A) and alkyl phosphonium (P) surfactants at a mass ratio of 1:1, 3:1 and 9:1 m/m, respectively, by ion exchange reaction, according to procedure described in the literature [13]. The organically modified purified montmorillonite (APO), with a mixture of different amounts of A and P surfactants, was coded as APOxA:yP where x:y is 1:1, 3:1, and 9:1, respectively.

### 3.3. PET Hybrids Preparation

To obtain the PET/APOxA:yP hybrids, we prepared concentrates (44 g of PET and 6 g of organophilic purified clay) by melt-mixing process, operating at 260 °C, 60 rpm for 10 min. Prior to processing, the PET polymer and the organoclays were dried for 6h at 160 °C and 60 °C, respectively. The concentrates obtained were mixed with PET polymer in quantities necessary to obtain a nominal content of 1%wt of organophilic purified montmorillonite (APOAxPy) by twin-screw extruder co-rotating, operating at 275 °C in all heating zones and with a rotation speed of 60 rpm. Then, the mixtures were processed by injection-molding (Arburg Allrounder Injection, Model M-250, Arburg, Frankfurt, Germany). The samples to tensile test were injected according to the ASTM D638, type I using the following processing conditions: temperature from 260, 270, 275, 280, and 285 °C in each heating zone.

### 3.4. Characterization

X-Ray diffraction: XRD analysis (LabX, Tokyo, Japan) was conducted on a Shimadzu XDR-6000 diffractometer with Cukα radiation (*λ* = 1.5418 Å), operating at 40 kV voltage and 30 mA current. The clays were analyzed as powder, while the PET/organoclays hybrids were analyzed as tensile specimens, Type I. All samples were scanned in 2*θ* ranges from 1.5 to 12° at a rate of 2.0°/min. With this technique it is possible to confirm the intercalation of the organic salts in the clay galleries by the expansion of basal spacing, and to investigate if a microcomposite or nanocomposite was produced.

The basal spacing (d001) value of bentonite was determined using Bragg’s law:

(1) $$d_{001}=\frac{\lambda }{2\sin\theta }$$

where *θ* is the measured diffraction angle in degree.

Thermogravimetric Analysis: TG (TA-60WS, Kyoto, Japan) analysis were conducted in a Shimadzu TGA S1H machine (Kyoto, Japan) employing approximately 15 mg of sample and platinum crucible and were heated from 25 to 900 °C at 10 °C/min in a flow of 50 mL/min under synthetic air atmosphere, with the purpose of analyzing the thermo-oxidative degradation of all samples.

Differential Scanning Calorimetry: DSC analyzes of pure PET and PET hybrids were conducted using a differential calorimeter, Perkin Elmer-model Pyris 6-DSC (Waltham, MA, United States), operating from 30 to 280 °C, maintained at 280 °C for 3 min, and then cooled to room temperature at a rate of 10 °C/min under nitrogen atmosphere. Then, these samples were heated again until 280 °C. The amount of sample used was approximately 5 mg, using aluminum crucible. The degree of crystallinity of the PET in the hybrids (Xc) was calculated using Equation (2).

(2) $$X_{c}(\%)=\frac{\Delta H_{m}}{\Delta H_{m}^{o}}\times 100$$

where $\Delta H_{m}^{o}$ is the heat of fusion of the PET sample studied (J/g) and $\Delta H_{m}^{o}$ is the heat of fusion for 100% crystalline PET ($\Delta H_{m}^{o}$ = 139.2 J/g) [36].

Tensile properties: The tensile test was conducted in a universal testing machine, Shimadzu-Autograph model—AG—IS, 100 KN (AG-X Plus Series, Shimadzu, Kyoto, Japan), employing a speed of 5 mm.min^−1^. The analysis of PET/organoclay hybrids were performed in the ASTM D638 (Type I). Six specimens of each hybrid were tested, and average values are presented in this paper.

Transmission Electron Microscopy (TEM): The TEM was used to evaluate the dispersion and the degree of intercalation and exfoliation of clay in the PET polymer. The TEM analyzes were performed in transmission electronic microscope of the Tecnai G2 (FEI Company, Hillsboro, USA) operating at an acceleration voltage at 200 kV. The samples were pressed into disc form with an average thickness of 1.5 mm after preparation on a Haake torque rheometer. The samples were prepared by reducing the area through the procedure of “trimmer” in trapezoidal shape with an area of approximately 0.5 mm^2^. The cuts of the samples were performed on a Leica ultramicrotome, model EM SCU—All Tech, using a diamond knife (Leica Microsystems, Wetzlar, Germany), with a cutting speed of 2 mm/s and a thickness of approximately 70 nm. The sections were placed on 300 mesh copper grids.

## 4. Conclusions

The incorporation of the mixture of surfactants, alkyl ammonium and phosphonium, resulted in obtaining thermally stable organoclays, as incorporation favored the obtaining of nanocomposites with intercalated and partially exfoliated morphologies. The intercalated morphology afforded a slight increase in the thermal stability as a result of the ordered structure of the PET nanocomposites. It can thus be concluded that the mixture of the salts is very promising in obtaining PET nanocomposites.

## Figures and Tables

**Table 1:** Weight loss of the clay AP and of the modified AP with a mixture of the surfactant A-P.

Samples	T_H_2_O_ (°C)	H_2_O (%)	T_max_ (°C)	Surfactant Loss (%)	Incorporated Surfactant (%)	Residue to 260 °C (%)
AP	71	10.25	-	-	-	-
APOA:P	49	1.31	275–302	18.50	68.39	3.71
APO3A:P	53	2.00	292	15.40	61.09	4.02
APO9A:P	58	2.59	282	16.34	70.12	4.34

T_H_2_O_—volatilization temperature of the water; H_2_O—percentage of water loss; T_max_—maximum decomposition temperature of the surfactant.

**Table 2:** Mass Loss of pure PET and PET hybrids with 1% by weight of the modified clay AP with the A-P salts mixture containing varying amounts of A and P.

Samples	T_max1_ (°C)	T_max2_ (°C)	T_D10%_ (°C)	Residues at 600 °C (%)
Pure PET	448	585	428	1.86
PET/APOA:P	450	558	432	3.17
PET/APO3A:P	449	564	435	2.73
PET/APO9A:P	445	559	429	3.17

T_max1_ and T_max2_—Maximum decomposition temperature belonging to the first and second stage of weight loss, respectively; T_D10%_—decomposition temperature at 10% of weight loss.

**Table 3:** Differential scanning calorimetry (DSC) data of pure PET and PET hybrids.

Samples	1° Heating		1° Cooling	2° Heating		
	Tg (°C)	T_ch_ (°C)	T_cc_ (°C)	Tm (°C)	ΔH_m_ (J/g)	Xc (%)
Pure PET	77	127	192	249	24.57	18.03
PET/APOA:P	77	127	191	249	31.05	22.80
PET/APO3A:P	76	125	188	249	7.56	25.55
PET/APO9A:P	77	126	194	249	41.27	30.30

Tg—Glass transition temperature; T_ch_—crystallizing temperature under heating; T_cc_—crystallization temperature on cooling; Tm—crystalline fusion temperature; ΔH_m_—fusion enthalpy; and Xc—degree of crystallinity.
